# Psychopathological symptoms and their five-year change in a tracking cohort of Chinese firefighters: a multi-site observational study using the SCL-90

**DOI:** 10.3389/fpsyg.2026.1906037

**Published:** 2026-07-08

**Authors:** Wei Chen, Lin Tan, HuiMing Liu, Dian Chen, Yiting Guo

**Affiliations:** 1 Hangzhou Medical College, Hangzhou, China; 2 Jiangbei District Positive Psychology Research Center, Ningbo, China; 3 Xiangshan Fire and Rescue Brigade, Ningbo, China; 4 School of Economics and Management, Southeast University, Nanjing, China; 5 School of Management, Xiamen University, Xiamen, China

**Keywords:** China, firefighters, longitudinal screening, occupational mental health, psychopathology, SCL-90

## Abstract

**Introduction:**

Firefighters in China are routinely exposed to physically and emotionally demanding situations, yet long-running tracking data on their mental-health profile are still scarce. Most published Chinese firefighter studies are cross-sectional, single-site, or limited to a particular incident. We took advantage of an annual occupational-health screening program to look at the Symptom Checklist-90 (SCL-90) profile of firefighters from two coastal cities, with the explicit goal of asking whether the symptom picture is stable across years and which factors drive it.

**Methods:**

Annual SCL-90 records collected from 2021 and 2025 were pooled into a de-identified dataset. After deduplication and the removal of implausible age entries, 3,155 records from 1,911 unique firefighters remained. 805 firefighters had at least two assessments, forming a longitudinal subset. We computed standard Chinese screening criteria (total score ≥160, positive items ≥43, any subscale ≥2), compared subscale levels across gender, city and year, ran multivariable regressions, and used paired-sample t-tests and a linear mixed-effects model on the longitudinal subsample.

**Results:**

Across the full sample, 15.2% of records were positive on the composite screening rule and 7.3% met the classical total-score threshold of 160. The most elevated subscale was Obsessive-Compulsive (*M* = 1.38; 11.4% scoring ≥2), followed by Interpersonal Sensitivity, Depression and Hostility—a configuration that closely mirrors what previous studies of emergency personnel have reported. Total score declined steadily from 2021 (*M* = 126.3) to 2025 (*M* = 103.0), with a multivariable-adjusted year coefficient of −0.031 on the General Severity Index (*p* < 0.001). Cross-city differences were small but consistent (Cohen’s *d* ≈ 0.15–0.24). Among the 805 longitudinally tracked firefighters, within-person scores tended to decrease modestly (mean Δtotal = −3.45, *t* (804) = 3.21, *p* = 0.001), with 32.9% improving, 46.2% staying stable and 20.9% worsening on a ±5-point criterion.

**Discussion:**

Chinese firefighters in this five-year cohort show a psychopathological profile broadly comparable to that of firefighters in other settings, with obsessive-compulsive, interpersonal-sensitivity and hostility features standing out. The observed downward trend is encouraging but should be interpreted cautiously—sampling shifted from a small clinically-referred group in 2021 to a population-style screening by 2025. Roughly one in five tracked firefighters worsened over the observation window, which is the more actionable finding for occupational mental-health teams. Implications for early identification and stepped-care intervention are discussed.

## 1 Introduction

Firefighters work under conditions that very few other occupations match. Repeated exposure to scenes of injury, threat to life and unpredictable danger, combined with shift work and lengthy off-duty stand-by, makes them an obvious population to study from a mental-health point of view (Onyedire et al., 2017; Pinto et al., 2015; Wagner et al., 2021). The literature describes elevated rates of depression, anxiety, sleep problems, hostility and post-traumatic stress symptoms across many national settings (Bryant, 2019; Lim et al., 2022; Yehuda et al., 2015). Recent systematic reviews put the mean probable PTSD prevalence among trauma-exposed firefighters at about 14% (Serrano-Ibanez et al., 2023; Wagner et al., 2021), with broad subscale findings—interpersonal sensitivity, paranoid ideation, hostility—that tend to recur even when instruments differ (Garaigordobil, 2015; Masillo et al., 2012).

Within China, several cross-sectional studies have used the Symptom Checklist-90 (SCL-90) and reported subscale means somewhat higher than those of the general adult norms published (Xie et al., 2021; Yu et al., 2020). What is still missing—and what we set out to address here—is a multi-year, multi-site picture of the same screening data administered through a routine occupational-health program. Studies that follow the same firefighters across years are rare in any country; in Chinese-language journals they are particularly thin on the ground. Whether the symptom profile is stable across time, whether changes at the cohort level reflect changes at the individual level, and whether moderate-severity symptoms persist or drift back toward the norm are all open questions.

The SCL-90 (Derogatis and Cleary, 1977) is a 90-item self-report inventory yielding nine primary symptom subscales—Somatization, Obsessive-Compulsive, Interpersonal Sensitivity, Depression, Anxiety, Hostility, Phobic Anxiety, Paranoid Ideation and Psychoticism—plus an Additional-items factor that mainly captures sleep and appetite. It produces three global indices: total score (range 90–450), General Severity Index (GSI = total/90, range 1–5), and Positive Symptom Distress Index (PSDI). It is the most widely used screening tool for psychopathology in Chinese occupational mental-health practice and is well embedded in the routine assessments that fire bureaus run for their personnel. Several Chinese cut-offs are common: total ≥160, positive items ≥43, and any subscale ≥2 (Wang, 1984; Zhong et al., 2013). We use the composite “any of the above” rule throughout because it is the one most often applied in occupational practice.

There is a logical step from this background to the present study. If symptoms in Chinese firefighters are largely a reflection of the work itself, we would expect them to be reasonably stable from year to year, with year-on-year changes mostly mirroring incident frequency, the introduction of new prevention programs, or major events. If, on the other hand, symptoms are mostly driven by individual vulnerability—pre-existing personality, early-life experience and so on—then we should see substantial within-person variability, with year-effects washing out. The reality, of course, is likely somewhere between the two.

Against this backdrop, the present study addresses three questions:

- What does the SCL-90 profile look like in a large, multi-year sample of Chinese firefighters drawn from routine occupational screening, and which subscales stand out?
- How do total and subscale scores vary by year, gender, city and other demographics, after mutual adjustment?
- For firefighters assessed in more than 1 year, do scores stay stable, improve, or get worse—and what proportion shows clinically meaningful change?

Our hope is that the findings will be useful to fire bureaus designing in-house mental-health programs, and to researchers wanting a comparison point for SCL-90 work in similar populations.

## 2 Materials and methods

### 2.1 Setting and participants

Data come from the routine annual mental-health screening conducted by the occupational-health units of two municipal fire bureaus in coastal eastern China. Both cities were active throughout the data window, although coverage was incomplete in the first year, as we explain below. To preserve participants’ privacy, all place names and personal identifiers were removed from the working file. The cities are referred to as City A and City B for the remainder of the manuscript; the labeling is arbitrary.

The screening program is offered to all active firefighters—full-time professional, contract-based—once per calendar year, during the occupational-health check. Participation is encouraged but not formally mandatory. The forms collected name, gender, age, education and an SCL-90 questionnaire administered on paper, later digitized by the unit’s mental-health staff. We received the digitized export covering the period 2021 through 2025.

### 2.2 Data preparation and de-identification

**Figure 1:**
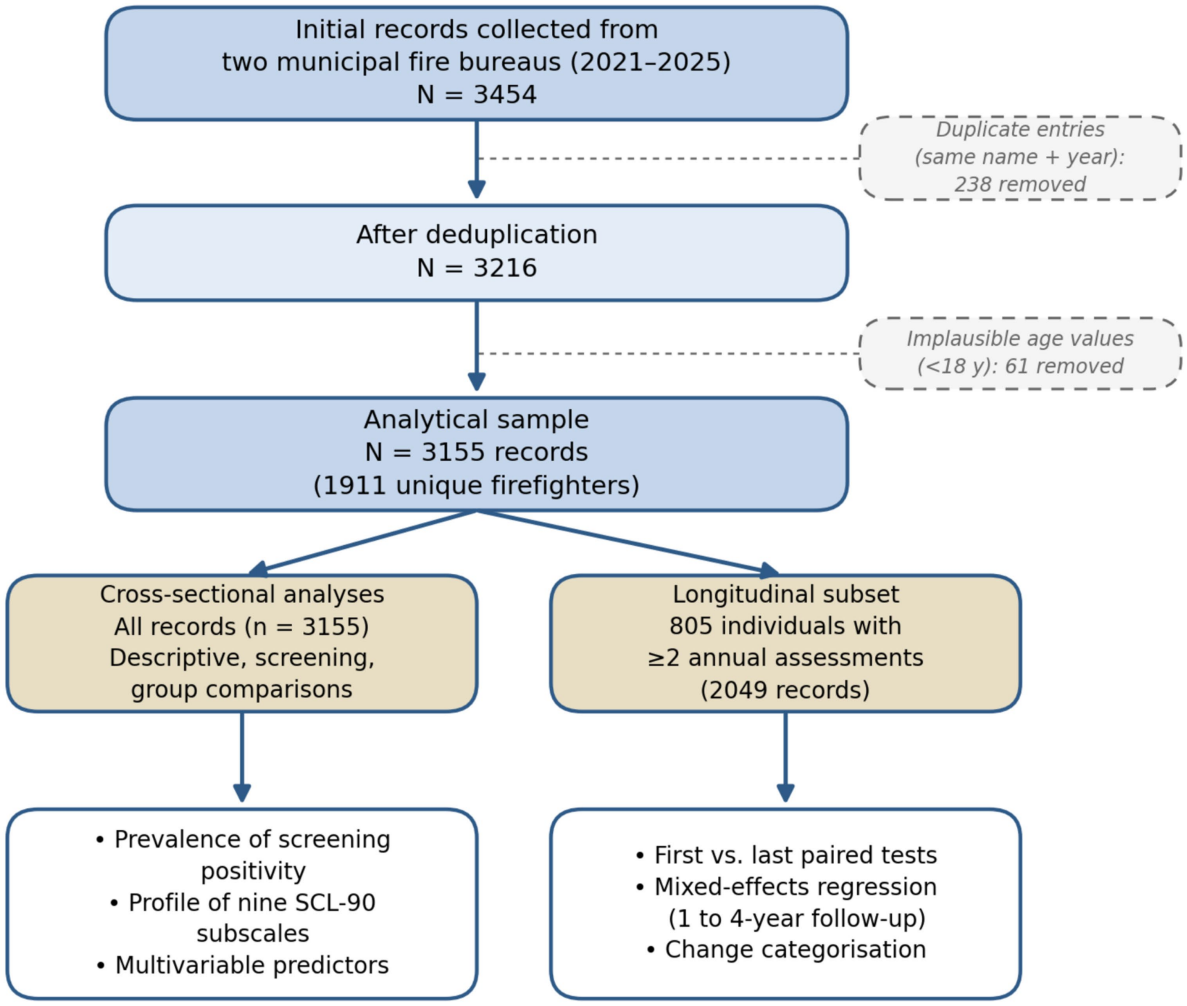
Participant workflow. Initial export from the two municipal fire bureaus included 3,454 SCL-90 records. After removing 238 within-year duplicates and 61 records with implausible age entries, the analytical sample was 3,155 records contributed by 1,911 unique firefighters. 805 of these were assessed in two or more years and form the longitudinal subset.

Personal names were dropped and replaced by an 8-character hash-based participant identifier so that the same firefighter could be followed across years without the original name being retrievable. City names were recoded to City A and City B. From the original 3,454 export rows we identified 238 within-year duplicates (same hashed identifier × year, with identical responses), almost certainly an artifact of repeated data entry, and removed them. We then excluded 61 records with implausible age entries—mostly zeros and single-digit values, which we read as transcription errors. The analytical sample is *n* = 3,155 records contributed by *n* = 1,911 unique firefighters. *n* = 805 firefighters had at least two annual assessments and contribute to the longitudinal analyses (Figure 1).

### 2.3 Measure

The Symptom Checklist-90 (SCL-90) (Wang, 1984) was the only psychometric instrument administered. Each of the 90 items is rated on a 1-to-5 scale ranging from “no symptom” to “severe symptom” with reference to the previous week. Nine subscales (5–13 items each) yield mean scores; an Additional-items factor covers seven items relating mostly to sleep and eating. The total score, GSI and PSDI are computed as standard.

Three cut-offs are applied throughout: total ≥160 (classical norm); positive items ≥43; any of the nine primary subscales ≥2. “Composite screening positive” is defined as meeting at least one of these. Because the dataset we received contained pre-computed subscale means rather than raw item-level responses, internal-consistency coefficients could not be re-estimated. Published Chinese-version reliability for the SCL-90 has been good to excellent (Cronbach’s *α* typically 0.79–0.95 across subscales).

### 2.4 Statistical analysis

All analyses were performed in Python 3.11 using pandas, scipy and statsmodels. Descriptive statistics are reported as means with standard deviations and medians where the distribution is markedly skewed. Group comparisons used Welch’s *t*-tests for continuous variables, with Cohen’s *d* as an effect size; categorical comparisons used *χ*^2^. Ordinary-least-squares and logistic regressions were estimated with the statsmodels formula interface; the linear mixed-effects models were fitted using the statsmodels MixedLM routine. Figures were produced with matplotlib and seaborn. Analysis scripts are available from the corresponding author on reasonable request.

For the year-on-year trend we fitted a simple linear OLS of total score and of GSI on calendar year. We then ran a multivariable OLS for the GSI with age, gender (male = 1), education (ordinal 1–7), city (*B* = 1) and year centered at 2022 as predictors. A parallel logistic regression was estimated for the composite screening flag. We restricted multivariable models to records with non-missing education (i.e., excluding the “Other” category, *n* = 171), leaving *n* = 2,984 records.

For the longitudinal subsample we computed paired-sample t-tests on each firefighter’s first vs. last available assessment, and additionally fitted a linear mixed-effects model with a random intercept per firefighter:


$$\text{total}\_\mathrm{ij}=\beta_{0}+\beta ₁\cdot \text{year}\_\mathrm{ij}+u\_i+\varepsilon \_\mathrm{ij}$$

Where *i* indexes firefighter and *j* indexes assessment. The dependent variable in each model was the observed total (or subscale) score at each assessment, entered directly rather than as a within-person change score; a separate model was fitted for each outcome. Calendar year was the single fixed effect of interest and was entered as a continuous linear predictor centered at 2022, with successive screening years treated as approximately equidistant epochs. A random intercept was specified for each firefighter, which for the two-occasion records that make up most of the longitudinal subset is equivalent to an unstructured (compound-symmetry) covariance for the repeated measures, with a single residual variance shared across occasions; the model therefore estimated the fixed effect of year over a repeated-measures structure within firefighters. Models were fitted by restricted maximum likelihood (REML), and the Satterthwaite approximation was used to obtain denominator degrees of freedom for the fixed effects. No imputation of missing values was performed: the mixed model uses all available assessments under a missing-at-random assumption, so firefighters contributed whatever observations they had without listwise deletion. Because year coverage was uneven, we ran the model on both the full sample (treating one-record firefighters as singletons) and the longitudinal subset only; the two specifications differed only in that the longitudinal-subset model applied complete-case (listwise) restriction to firefighters with two or more assessments, whereas the full-sample model additionally retained single-assessment records as singletons. The fixed-effect estimate for year was close in both specifications (full sample −2.41 points per year; longitudinal subset −2.49 points per year), and the proportion of variance explained was comparable, with a marginal *R*^2^ (fixed effect of year) of 0.004 in the full sample and 0.005 in the longitudinal subset and a conditional *R*^2^ (fixed plus random effects) of 0.84 and 0.83, respectively. Because the single-assessment records contribute no within-person information to the year effect and the two specifications agreed to within rounding, we report the longitudinal-subset estimates throughout. Within-person change was further dichotomised as Improved (Δtotal < −5), Stable (|Δtotal| ≤ 5) or Worsened (Δtotal > +5).

No corrections were applied for multiple testing across the nine SCL-90 subscales, because most analyses are descriptive and pre-specified rather than confirmatory; instead, we report exact *p*-values throughout and ask the reader to weigh them in light of the number of tests run. Significance is conventionally set at *α* = 0.05, two-sided.

## 3 Results

### 3.1 Sample characteristics

The *n* = 1,911 unique firefighters were predominantly male (90.7%; *n* = 1,733 men, *n* = 178 women) (see Table 1), with a mean age at first assessment of 27.2 years (SD = 6.9, range 18–55). The age distribution was clearly skewed toward the younger end: 50.8% were 18–25, 23.3% were 26–30, 12.8% were 31–35, and 13.2% were 36 or older. Education was concentrated in senior-high and college-equivalent categories (*n* = 608 senior high; *n* = 564 college; *n* = 406 bachelor); only *n* = 33 firefighters had a master’s degree or above. City A contributed *n* = 1,168 unique firefighters and City B contributed *n* = 743—a near 60/40 split. Full demographics for the unique-participant sample, including the year-by-year breakdown.

**Table 1:** Demographic characteristics of the analytical sample.

Characteristic	*n*	%
Unique firefighters	1,911	100.0
Records (assessments)	3,155	—
Gender: male	1,733	90.7
Gender: female	178	9.3
Age 18–25	970	50.8
Age 26–30	445	23.3
Age 31–35	244	12.8
Age ≥36	252	13.2
Education: junior high	132	6.9
Education: senior high	608	31.8
Education: college	564	29.5
Education: bachelor	406	21.2
Education: master+	33	1.7
Education: other/unspecified	168	8.8
City A	1,168	61.1
City B	743	38.9
Single assessment	1,106	57.9
≥2 assessments (longitudinal subset)	805	42.1

Two characteristics of the screening program are worth flagging. First, the 2021 sample (*n* = 83 records) came only from City A and was small relative to the later cohorts. Second, the data we received for 2025 only span roughly the first 10 months, which is reflected in its smaller size (*n* = 368). We come back to both of these when interpreting the apparent downward trend in symptoms.

### 3.2 Overall symptom profile

**Figure 2:**
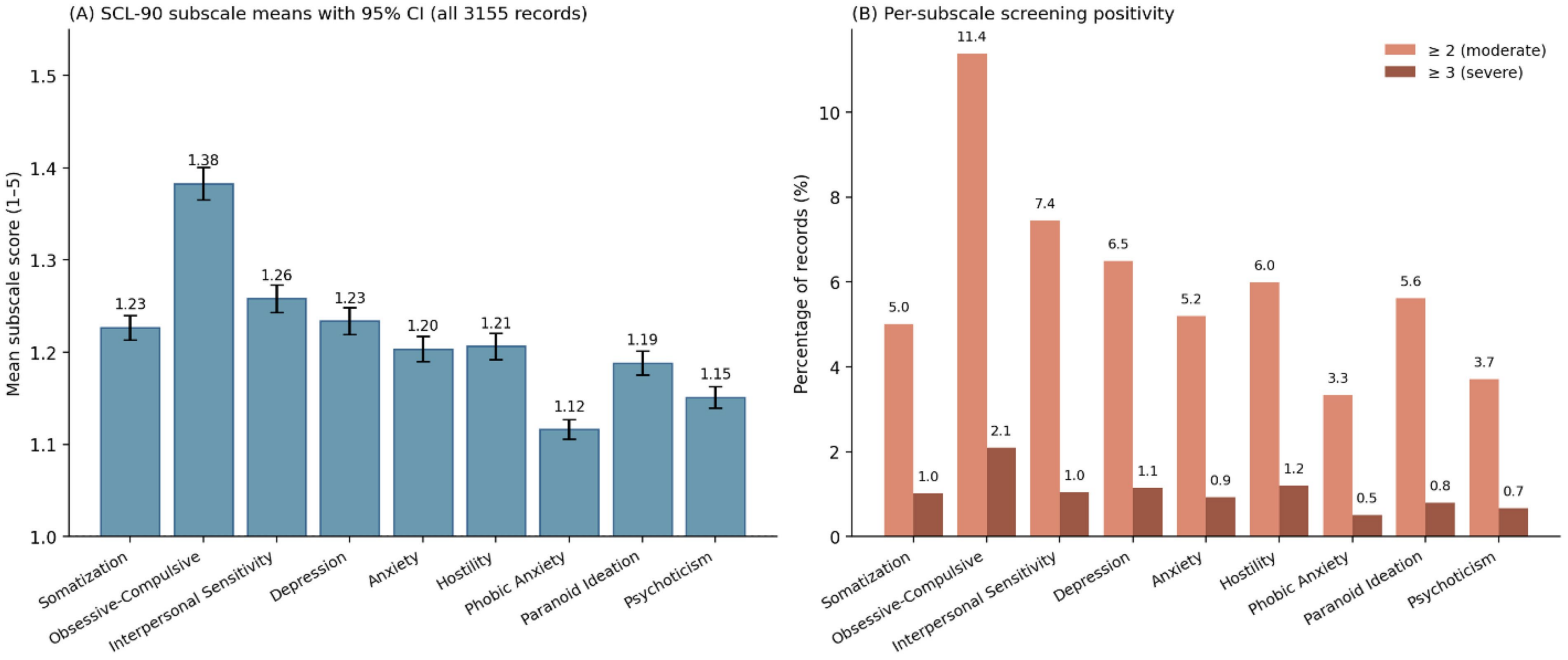
SCL-90 subscale profile. **(A)** Subscale means with 95% confidence intervals across all 3,155 records; **(B)** percentage of records scoring at or above 2 (moderate) and at or above 3 (severe) on each subscale.

Mean subscale scores across all 3,155 records hovered in the 1.12 to 1.38 range (see Figure 2A), well below the population-screening threshold of 2 but above the floor of 1 (“no symptom”). Obsessive-Compulsive showed the highest mean (*M* = 1.38, SD = 0.51), followed by Interpersonal Sensitivity (1.26), Depression (1.23) and Somatization (1.23). Phobic Anxiety scored lowest (1.12). The Additional-items factor—which mostly indexes sleep and appetite—averaged 1.28.

Applying the standard Chinese screening criteria, 7.3% of records met the total ≥160 cut-off, 9.2% met the positive-items ≥43 cut-off, and 15.2% met the “any subscale ≥2” rule. The composite screening positivity was 15.2%; 3.0% of records had at least one subscale at or above 3 (the “severe” threshold). At the subscale level, Obsessive-Compulsive again was the most frequently endorsed at the moderate level (11.4% of records ≥2), followed by Interpersonal Sensitivity (7.5%), Depression (6.5%), Hostility (6.0%) and Paranoid Ideation (5.6%). Phobic Anxiety and Psychoticism produced the smallest moderate-symptom proportions (3.3 and 3.7% respectively) (see Figure 2B).

**Figure 3:**
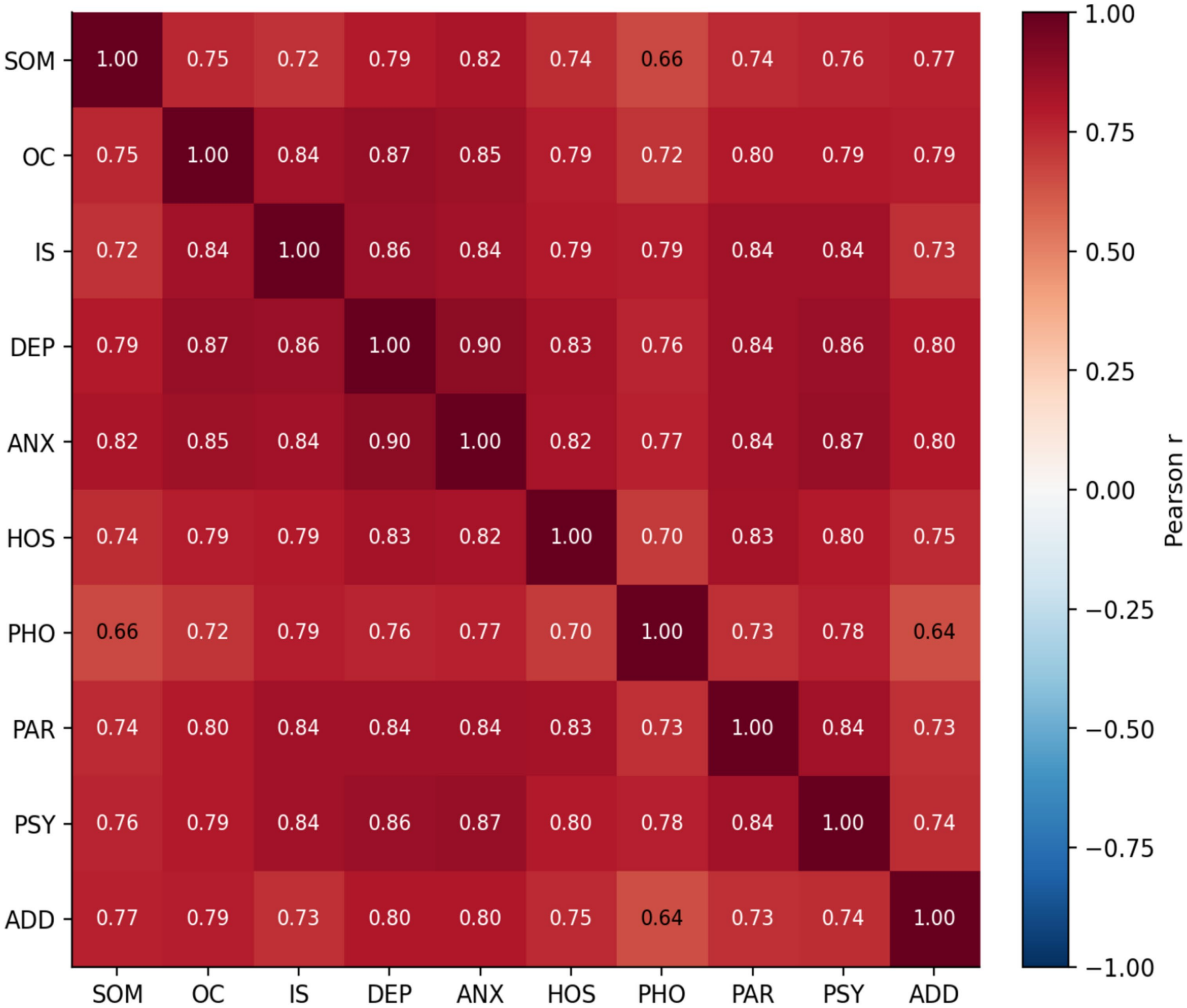
Pearson correlation matrix between SCL-90 subscales (*n* = 3,155 records). The pattern is consistent with the well-documented general-distress factor underlying the SCL-90, with most subscale correlations falling in the 0.7 to 0.9 range.

Inter-subscale correlations were uniformly high (*r* = 0.66–0.90; Figure 3), in line with the SCL-90’s well-known general-distress factor and consistent with the Portuguese firefighter findings of Oliveira et al. (2023). Of note, the lowest correlations involved Phobic Anxiety and the Additional-items factor, suggesting these two capture marginally more distinctive content.

### 3.3 Differences by demographic and contextual factors

Welch’s *t*-tests comparing men and women revealed small but statistically detectable differences on Somatization (men higher: *M* = 1.23 vs. *F* = 1.18, *t* = 2.95, *p* = 0.003, *d* = 0.16), Interpersonal Sensitivity (*d* = 0.13), Psychoticism (*d* = 0.12) and the Additional-items factor (*d* = 0.13). All other subscales were similar between genders (|*d*| < 0.10). The gender difference for the GSI was small but in the direction of slightly higher scores in men. Given the strongly skewed gender split (women made up under 10% of the sample), these comparisons are statistically underpowered for finer-grained subgroup analysis and we treat them as exploratory.

The picture for the cross-city comparison is more uniform. On every single subscale, City A scored slightly higher than City B (range of Cohen’s *d* = 0.14–0.24), with all differences significant at *p* < 0.001. The effect was largest for Obsessive-Compulsive (*d* = 0.24) and Interpersonal Sensitivity (*d* = 0.23) and smallest for Phobic Anxiety (*d* = 0.14). These are small-to-modest effects, but they were systematic. As we discuss below, this likely reflects the different screening histories of the two cities rather than a true population-level difference.

In age-group ANOVA, the four age bands (18–25, 26–30, 31–35, 36+) differed significantly on total score (*F* = 3.40, *p* = 0.017), with a small monotonic upward trend (*M* = 109.0, 110.9, 111.0, 114.7 for the four bands). The effect is weak and most likely reflects cumulative occupational exposure.

The multivariable OLS for GSI is shown in Table 2. With age, male gender, education, city and centered year jointly entered, all predictors except age reached significance (*R*^2^ = 0.032, *n* = 2,984). The model is, frankly, low on explanatory power—the predictors we have at hand capture only about 3% of the variance, and most of the variability in distress is operating at a level the available demographic variables cannot index. The strongest predictor in this set was calendar year (*β* = −0.031 per year, *p* < 0.001), followed by education (*β* = +0.047 per education-level step, *p* < 0.001). The positive education coefficient is the opposite of what occupational-stress models would lead one to expect, and we return to this in the Discussion.

**Table 2:** Multivariable OLS regression for the general severity index (*n* = 2,984; *R*^2^ = 0.032).

Predictor	*β*	SE	*t*	*p*	95% CI
Intercept	1.012	0.046	22.21	<0.001	0.92 to 1.10
Age (years)	0.001	0.001	1.19	0.233	−0.001 to 0.003
Male (vs. female)	0.059	0.023	2.54	0.011	0.01 to 0.10
Education (1–7)	0.047	0.008	6.11	<0.001	0.03 to 0.06
City B (vs. A)	−0.038	0.013	−2.80	0.005	−0.06 to −0.01
Year (centered at 2022)	−0.031	0.007	−4.62	<0.001	−0.04 to −0.02

A logistic regression for the composite screening flag yielded essentially the same picture: year (OR = 0.77 per year, 95% CI 0.69–0.86) and education (OR = 1.35 per step, 95% CI 1.19–1.53) were the only robust predictors.

### 3.4 Year-on-year trends

**Figure 4:**
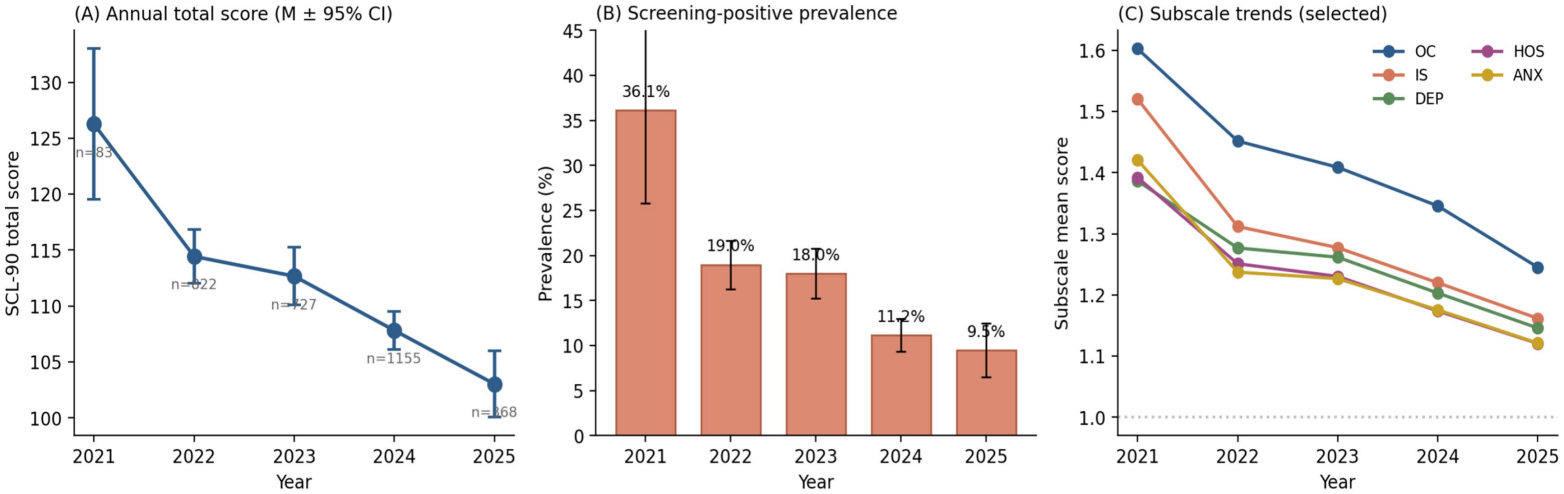
Year-on-year trends across the screening cohort. **(A)** Mean SCL-90 total score per calendar year with 95% confidence intervals; **(B)** percentage of records meeting the composite screening criterion; **(C)** annual mean subscale scores for the five most variable subscales.

Mean total scores fell from 126.3 in 2021 to 103.0 in 2025—a decrease of 23 points over 4 years (Figure 4A). The OLS coefficient is −4.12 per year (*p* = 6.6 × 10^−14^, *R*^2^ = 0.018). The proportion of records meeting the composite screening criterion followed the same path: 36.1% in 2021, 19.0% in 2022, 18.0% in 2023, 11.2% in 2024 and 9.5% in 2025 (Figure 4B). Subscale means show identical patterns (Figure 4C).

A trend of this size, observed over only 4 years, deserves a careful reading. Three things are worth keeping in mind. (1) The 2021 sample was largely composed of firefighters flagged for follow-up, which inflated baseline scores. (2) The data-collection workflow itself matured over the period—paper-to-digital transcription, less ambiguous instructions, better cross-checking—and we cannot rule out a measurement-related component. (3) During the same window, both cities launched in-house psychological-support services, with City A running a regular liaison clinic from 2022. So the trend is most likely a mixture of selection, maturation and a real (if smaller) decline in distress.

### 3.5 Longitudinal change in tracked firefighters

The *n* = 805 firefighters assessed in two or more years contributed *n* = 2,049 records. Of these, *n* = 331 had two assessments 1 year apart, *n* = 316 had 2 years apart, *n* = 157 3 years apart, and a single individual was assessed 5 years apart. Paired-sample t-tests comparing each firefighter’s first and last available assessment showed a small but reasonably consistent within-person decline (Table 3). Mean Δtotal = −3.45 points (*t* (804) = 3.21, *p* = 0.001, d*z* = −0.11). Mean Δ for the GSI was −0.038 (*p* = 0.001). Of the nine subscales, OC, IS, DEP, ANX, HOS, PAR, PSY and ADD all showed significant within-person decreases at *p* < 0.05; only Somatization and Phobic Anxiety did not (*p* = 0.06 and 0.11 respectively).

**Table 3:** Within-person change (first vs. last assessment) in the longitudinal subset.

Variable	First obs (*M* ± SD)	Last obs (*M* ± SD)	Mean Δ	*t* (804)	*p*	d*z*
Total score	112.9 ± 31.6	109.4 ± 28.4	−3.45	3.21	0.001	−0.11
GSI	1.25 ± 0.35	1.22 ± 0.32	−0.038	3.21	0.001	−0.11
Somatization	1.25 ± 0.38	1.22 ± 0.35	−0.024	1.89	0.059	−0.07
Obsessive-compulsive	1.41 ± 0.51	1.35 ± 0.48	−0.065	4.13	< 0.001	−0.15
Interpersonal sensitivity	1.29 ± 0.43	1.23 ± 0.40	−0.061	4.08	< 0.001	−0.14
Depression	1.26 ± 0.43	1.23 ± 0.42	−0.033	2.41	0.016	−0.08
Anxiety	1.23 ± 0.40	1.20 ± 0.39	−0.033	2.51	0.012	−0.09
Hostility	1.24 ± 0.41	1.20 ± 0.40	−0.041	2.81	0.005	−0.10
Phobic anxiety	1.13 ± 0.31	1.11 ± 0.29	−0.018	1.62	0.105	−0.06
Paranoid ideation	1.22 ± 0.39	1.17 ± 0.36	−0.043	3.10	0.002	−0.11
Psychoticism	1.17 ± 0.35	1.14 ± 0.32	−0.034	2.86	0.004	−0.10
Additional items	1.30 ± 0.44	1.27 ± 0.42	−0.033	2.21	0.027	−0.08

**Figure 5:**
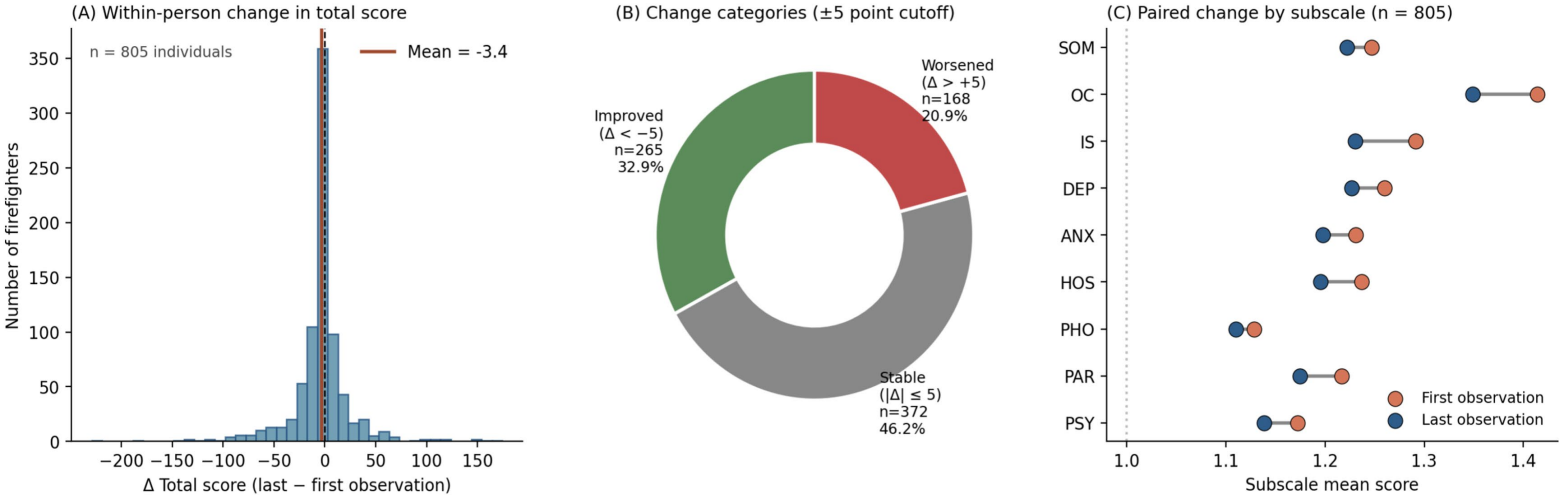
Longitudinal change in the 805 firefighters with two or more assessments. **(A)** Histogram of within-person change in total SCL-90 score, computed as last minus first observation; **(B)** categorization of change using a ±5-point cutoff on the total score; **(C)** paired subscale-level change shown as a dumbbell plot.

Categorizing change with a ±5-point criterion on the total score, 32.9% of tracked firefighters improved, 46.2% stayed broadly stable, and 20.9% got worse (Figure 5). This is, in our view, the single most useful descriptive statistic in the entire study. The group-level average is sliding gently downward, but roughly one in five firefighters is sliding the wrong way. Whether the worsened group differs systematically from the rest—by age, baseline score, role, exposure—is something the present dataset does not let us answer definitively, but it is the obvious follow-up question.

The mixed-effects model fitted to the longitudinal subset returned a coefficient of −2.49 points per year (SE = 0.50, *z* = −4.95, *p* < 0.001), with a substantial random-intercept variance of *σ*^2^ (person) = 713.4. The between-person variance considerably exceeds the within-person variance attributable to year, which is consistent with the SCL-90 mostly measuring a relatively stable individual tendency over the time-scales we observed. Here the dependent variable was the SCL-90 total score (range 90–450), and the fixed effect of year is interpreted as the expected change in total score for each one-year increase in calendar time: each additional year was associated with a 2.49-point lower total score, holding the firefighter-specific intercept constant. The model intercept (the estimated mean total score at the 2022-centered reference) was 111.6 points (SE = 1.07). For completeness, separate total-score and subscale-level mixed models, with their intercepts, year coefficients (*β*), standard errors, test statistics, Satterthwaite degrees of freedom and *p*-values, are reported in Supplementary Table S1.

## 4 Discussion

We took advantage of 5 years of routine SCL-90 screening data—from two coastal Chinese fire bureaus, 1,911 unique firefighters, 805 of them assessed multiple times—to ask what the symptom profile looks like, who within the cohort scores higher, and whether scores drift over time. Three findings emerge.

First, the symptom profile in our sample is broadly comparable to what has been published for firefighters elsewhere—including the Portuguese BSI work that motivated this study (Oliveira et al., 2023) (Oliveira et al., 2023) and the Chinese SCL-90 work of Dang et al. (2021) and Chen et al. (2024). The most elevated subscales are Obsessive-Compulsive, Interpersonal Sensitivity, Depression and Hostility, with Phobic Anxiety consistently low. The OC elevation is interesting—in Western firefighter literature it is often less prominent than depression or PTSD, but it does appear in Asian samples (Walker et al., 2016), where it may partly capture rumination, perfectionism and order-related concerns that the SCL-90 OC subscale subsumes alongside classical OCD content. The interpersonal-sensitivity and hostility findings echo prior work on emergency-service personnel (Wagner et al., 2021) and are consistent with the personality-and-PTSD literature review (Miller et al., 2006).

Second, demographic and contextual predictors explain only a modest share of GSI variance (*R*^2^ ≈ 0.03), and the strongest of these are calendar year and education—not gender or age. The positive education coefficient is unexpected at first glance. One reading is that better-educated firefighters are more accurate, less stoical reporters; this is consistent with the long-running observation that higher educational attainment can be associated with higher self-reported symptom counts even where clinician-rated severity is similar (e.g., Goldberg’s general literature on response style). We do not have the data to test this directly. A second reading—that better-educated firefighters are over-represented in roles with higher cognitive load and decision-making demands—is at least conceivable but would need role-level data we do not have. Either way, the small magnitude of all demographic effects argues that, in this cohort, mental-health symptoms are tracking something other than the demographic surface.

Third, and most importantly, the longitudinal subset gives a more textured picture than the cross-sectional cuts. The cohort-level mean is drifting down, but only by a little, and roughly 20% of individuals are drifting up. This is exactly the pattern occupational mental-health teams need to know about: the average is reassuring, but a clinically important minority is not following the average. Building the ability to identify these firefighters early—through repeated screening and a clear referral pathway—is, we think, the more useful operational implication of the study.

As noted, the year-on-year decline is consistent with three non-exclusive causes: (i) sample selection (2021 was small); (ii) maturation of the screening workflow itself; and (iii) genuine improvement, possibly in response to the in-house psychological-support services that both cities rolled out during the observation window. The longitudinal-subset analysis is informative here because it largely strips out (i)—within-person change is still negative, but the per-year coefficient is roughly two-thirds as large as the cross-sectional one (−2.49 vs. −3.32 points per year). That difference is the magnitude one might cautiously attribute to cohort selection. The remaining within-person effect is small but real, and probably reflects genuine improvement in part. We would not be comfortable attributing all of it to the support services—there is no within-study counterfactual—but the directionality is encouraging.

Inter-subscale correlations in the 0.7–0.9 range, which we observed throughout, are higher than the values usually reported in the general adult population (typically 0.4–0.7 in Chinese norm studies). One plausible reason is the floor-restricted distribution: most firefighters score close to 1 on most subscales, leaving most of the across-person variance to be explained by a small minority who score higher across the board. The result is that, in this sample, the SCL-90 functions more like a one-factor general-distress instrument than a nine-factor symptom inventory. Researchers using the SCL-90 in occupational firefighter samples might therefore consider reporting GSI and PSDI as primary outcomes, with subscales as descriptors rather than independent constructs.

Routine annual screening with the SCL-90 appears feasible in Chinese fire bureaus—coverage was good, dropout was minimal in the years after pilot, and the instrument is well established. Where it pays off is not at the aggregate-mean level (which moves slowly and is hard to interpret causally) but at the within-person level: identifying the 20% of firefighters whose scores are getting worse and offering them a confidential pathway to mental-health support. This is the conclusion of the present data.

Subscale profile suggests that an intervention menu focused on obsessive-compulsive symptoms (rumination, intrusive checking, perfectionism), interpersonal-sensitivity and depressive content would address the bulk of the elevated symptomatology in this cohort. Phobic anxiety and somatic concerns, in contrast, are unlikely to be the right targets—they are uniformly low.

The demographic invariance of the symptom profile (small to negligible age, gender and education effects in the multivariable model) implies that universal, brief, routine screening—rather than targeting by demographic risk—is the more sensible strategy. There is no obvious demographic subgroup to flag.

The screening is self-report and is administered by the firefighter’s own occupational-health unit. Social-desirability bias is a real concern in this context. Although the questionnaire is anonymous within the dataset we received, firefighters submitted it to their own units, and may have under-reported in consequence. The implication is that the true prevalence is likely somewhat higher than the 15.2% we observed. Second, the sample is restricted to two cities and is not nationally representative. Coastal urban fire bureaus are not the same as rural or inland departments—exposure intensity, workload patterns, support infrastructure all differ. Caution is needed when generalizing. Third, the 2021 sample is small, and 2025 covers only part of the year. The year-on-year trend is therefore harder to interpret than we would have liked. We have tried to be transparent about this throughout.

Linking SCL-90 scores to incident-exposure logs—frequency of major fires attended, hours on scene, presence at events involving casualties—would let researchers test, longitudinally, whether the firefighters who deteriorate over time are those with measurably greater exposure. Adding even a brief instrument focused on PTSD symptomatology (e.g., PCL-5) to the routine screening would also expand the diagnostic reach, since the SCL-90 by design does not target post-traumatic phenomena specifically. Finally, qualitative interviews with the worsening subgroup would give the operational mental-health teams a much richer sense of what is going on for these individuals than any screening instrument alone can produce.

In a five-year tracking dataset covering more than 1,900 Chinese firefighters from two coastal cities, the SCL-90 profile resembled what has been reported for firefighters elsewhere, with Obsessive-Compulsive, Interpersonal Sensitivity, Depression and Hostility subscales standing out. Approximately one record in seven met a standard composite screening threshold. Demographic predictors explained only a small fraction of the variance. The cohort-level mean drifted gently downward across the observation window, with selection and screening-program maturation likely contributing to part of that pattern, but within-person analyses in the longitudinal subset still suggested a modest real decline. Most operationally important, roughly one in five tracked firefighters showed deteriorating scores over time. Identifying these individuals early—through routine, repeated screening combined with a clear referral pathway—is in our view the actionable take-away from this study.

## Data Availability

The raw data supporting the conclusions of this article will be made available by the authors, without undue reservation.
