# Supplementary material for: Psychopathological symptoms and their five-year change in a tracking cohort of Chinese firefighters: a multi-site observational study using the SCL-90
Source: Front Psychol. 2026 Jul 8;17:1906037. doi: 10.3389/fpsyg.2026.1906037 (PMC13388276; doi:10.3389/fpsyg.2026.1906037)
Supplement: Supplementary file 1 [file Table_1.DOCX]

**Supplementary Material**

*for the manuscript: "Psychopathological symptoms and their five-year change in a tracking cohort of Chinese firefighters: a multi-site observational study using the SCL-90"*

**Supplementary Table S1. Detailed sample characteristics**

All counts and percentages refer to unique participants (n = 1,911) computed from each firefighter's first available assessment.

| **Characteristic** | **n / Value** | **%** |
| --- | --- | --- |
| Total unique participants | 1911 | 100.0% |
| Total records | 3155 | — |
| Gender |  |  |
| Male | 1733 | 90.7% |
| Female | 178 | 9.3% |
| Age (years) | M = 27.2, SD = 6.9 | range 18–55 |
| 18–25 | 970 | 50.8% |
| 26–30 | 445 | 23.3% |
| 31–35 | 244 | 12.8% |
| ≥ 36 | 252 | 13.2% |
| Education |  |  |
| SeniorHigh | 608 | 31.8% |
| College | 564 | 29.5% |
| Bachelor | 406 | 21.2% |
| Other | 167 | 8.7% |
| JuniorHigh | 132 | 6.9% |
| Master | 32 | 1.7% |
| Primary | 1 | 0.1% |
| Doctorate | 1 | 0.1% |
| City (anonymised) |  |  |
| City A | 1168 | 61.1% |
| City B | 743 | 38.9% |
| Records per participant |  |  |
| 1 year(s) | 1106 | 57.9% |
| 2 year(s) | 451 | 23.6% |
| 3 year(s) | 270 | 14.1% |
| 4 year(s) | 83 | 4.3% |
| 5 year(s) | 1 | 0.1% |

**Supplementary Table S2. Subscale descriptive statistics**

Computed across all 3,155 records. % ≥ 2 indicates the proportion of records meeting the at-least-moderate threshold on each subscale; % ≥ 3 indicates the at-least-severe threshold.

| **Subscale** | **Mean** | **SD** | **Median** | **Range** | **% ≥ 2** | **% ≥ 3** |
| --- | --- | --- | --- | --- | --- | --- |
| Somatization | 1.227 | 0.382 | 1.08 | 1.00–5.00 | 5.01 | 1.01 |
| Obsessive-Compulsive | 1.383 | 0.506 | 1.20 | 1.00–5.00 | 11.38 | 2.09 |
| Interpersonal Sensitivity | 1.258 | 0.433 | 1.11 | 1.00–5.00 | 7.45 | 1.05 |
| Depression | 1.234 | 0.423 | 1.08 | 1.00–5.00 | 6.50 | 1.14 |
| Anxiety | 1.203 | 0.388 | 1.00 | 1.00–5.00 | 5.20 | 0.92 |
| Hostility | 1.206 | 0.406 | 1.00 | 1.00–5.00 | 5.99 | 1.20 |
| Phobic Anxiety | 1.116 | 0.304 | 1.00 | 1.00–5.00 | 3.33 | 0.51 |
| Paranoid Ideation | 1.188 | 0.376 | 1.00 | 1.00–5.00 | 5.61 | 0.79 |
| Psychoticism | 1.151 | 0.335 | 1.00 | 1.00–5.00 | 3.71 | 0.67 |
| Additional Items | 1.278 | 0.435 | 1.14 | 1.00–5.00 | 8.24 | 1.27 |

**Supplementary Table S3. Year-by-year summary**

Coverage of the 2021 wave was limited to a small group from City A; the 2025 wave only covers approximately the first ten months of the calendar year. These differences contribute to the apparent year-on-year decline in mean scores discussed in the main text.

| **Year** | **N** | **Total score (M ± SD)** | **GSI** | **Composite screening +** | **Total ≥ 160** |
| --- | --- | --- | --- | --- | --- |
| 2021 | 83 | 126.3±31.3 | 1.403 | 30 (36.1%) | 17 (20.5%) |
| 2022 | 822 | 114.4±35.3 | 1.271 | 156 (19.0%) | 73 (8.9%) |
| 2023 | 727 | 112.7±35.6 | 1.252 | 131 (18.0%) | 60 (8.3%) |
| 2024 | 1155 | 107.8±29.2 | 1.198 | 129 (11.2%) | 66 (5.7%) |
| 2025 | 368 | 103.0±29.2 | 1.144 | 35 (9.5%) | 15 (4.1%) |

**Supplementary Table S4. Group comparisons (Welch's t)**

Effect sizes were computed as Cohen's d using the pooled within-group standard deviation. Positive d values for the gender comparison favour men (higher symptom score); positive d values for the city comparison favour City A.

| **Subscale** | **Comparison** | **Group 1 (M±SD)** | **Group 2 (M±SD)** | **t** | **p** | **Cohen's d** |
| --- | --- | --- | --- | --- | --- | --- |
| Somatization | Gender (M vs F) | 1.232±0.389 | 1.175±0.301 | 2.95 | 0.0034 | 0.164 |
| Obsessive-Compulsive | Gender (M vs F) | 1.381±0.510 | 1.405±0.460 | -0.84 | 0.4004 | -0.050 |
| Interpersonal Sensitivity | Gender (M vs F) | 1.263±0.439 | 1.212±0.366 | 2.16 | 0.0314 | 0.125 |
| Depression | Gender (M vs F) | 1.233±0.426 | 1.243±0.383 | -0.43 | 0.6700 | -0.025 |
| Anxiety | Gender (M vs F) | 1.205±0.396 | 1.187±0.299 | 0.93 | 0.3551 | 0.051 |
| Hostility | Gender (M vs F) | 1.205±0.409 | 1.218±0.373 | -0.55 | 0.5848 | -0.033 |
| Phobic Anxiety | Gender (M vs F) | 1.118±0.308 | 1.104±0.256 | 0.82 | 0.4128 | 0.047 |
| Paranoid Ideation | Gender (M vs F) | 1.189±0.381 | 1.176±0.326 | 0.65 | 0.5155 | 0.038 |
| Psychoticism | Gender (M vs F) | 1.154±0.344 | 1.120±0.225 | 2.30 | 0.0222 | 0.118 |
| Additional Items | Gender (M vs F) | 1.282±0.443 | 1.232±0.338 | 2.31 | 0.0215 | 0.128 |
| Somatization | City (A vs B) | 1.264±0.398 | 1.182±0.358 | 6.11 | 0.0000 | 0.217 |
| Obsessive-Compulsive | City (A vs B) | 1.437±0.518 | 1.318±0.485 | 6.61 | 0.0000 | 0.236 |
| Interpersonal Sensitivity | City (A vs B) | 1.303±0.453 | 1.205±0.402 | 6.45 | 0.0000 | 0.229 |
| Depression | City (A vs B) | 1.264±0.427 | 1.198±0.414 | 4.43 | 0.0000 | 0.158 |
| Anxiety | City (A vs B) | 1.238±0.405 | 1.162±0.362 | 5.56 | 0.0000 | 0.198 |
| Hostility | City (A vs B) | 1.242±0.433 | 1.163±0.367 | 5.55 | 0.0000 | 0.197 |
| Phobic Anxiety | City (A vs B) | 1.135±0.321 | 1.094±0.281 | 3.89 | 0.0001 | 0.138 |
| Paranoid Ideation | City (A vs B) | 1.218±0.390 | 1.153±0.355 | 4.88 | 0.0000 | 0.174 |
| Psychoticism | City (A vs B) | 1.177±0.351 | 1.120±0.312 | 4.87 | 0.0000 | 0.173 |
| Additional Items | City (A vs B) | 1.317±0.454 | 1.231±0.406 | 5.56 | 0.0000 | 0.198 |

**Supplementary Table S5. Inter-subscale Pearson correlations**

All correlations are computed across the 3,155-record analytical sample. Subscales share substantial common variance, consistent with the well-documented general-distress factor underlying the SCL-90.

|  | **SOM** | **OC** | **IS** | **DEP** | **ANX** | **HOS** | **PHO** | **PAR** | **PSY** | **ADD** |
| --- | --- | --- | --- | --- | --- | --- | --- | --- | --- | --- |
| SOM | 1.0 | 0.753 | 0.724 | 0.794 | 0.819 | 0.74 | 0.659 | 0.742 | 0.759 | 0.77 |
| OC | 0.753 | 1.0 | 0.843 | 0.872 | 0.85 | 0.788 | 0.715 | 0.796 | 0.794 | 0.785 |
| IS | 0.724 | 0.843 | 1.0 | 0.861 | 0.839 | 0.791 | 0.785 | 0.843 | 0.837 | 0.733 |
| DEP | 0.794 | 0.872 | 0.861 | 1.0 | 0.898 | 0.829 | 0.761 | 0.842 | 0.859 | 0.802 |
| ANX | 0.819 | 0.85 | 0.839 | 0.898 | 1.0 | 0.824 | 0.77 | 0.839 | 0.873 | 0.802 |
| HOS | 0.74 | 0.788 | 0.791 | 0.829 | 0.824 | 1.0 | 0.703 | 0.83 | 0.796 | 0.746 |
| PHO | 0.659 | 0.715 | 0.785 | 0.761 | 0.77 | 0.703 | 1.0 | 0.733 | 0.781 | 0.643 |
| PAR | 0.742 | 0.796 | 0.843 | 0.842 | 0.839 | 0.83 | 0.733 | 1.0 | 0.84 | 0.727 |
| PSY | 0.759 | 0.794 | 0.837 | 0.859 | 0.873 | 0.796 | 0.781 | 0.84 | 1.0 | 0.741 |
| ADD | 0.77 | 0.785 | 0.733 | 0.802 | 0.802 | 0.746 | 0.643 | 0.727 | 0.741 | 1.0 |

**Supplementary Table S6. Within-person change (longitudinal subset)**

Paired t-tests comparing each firefighter's first and last available SCL-90 assessment in the longitudinal subset (n = 805 firefighters with two or more assessments). Negative Δ values indicate within-person decreases in symptom severity over time.

| **Variable** | **First obs (M±SD)** | **Last obs (M±SD)** | **Mean Δ** | **t** | **p** | **Cohen dz** |
| --- | --- | --- | --- | --- | --- | --- |
| Somatization | 1.247±0.383 | 1.222±0.393 | -0.024 | -1.89 | 0.0594 | -0.067 |
| Obsessive-Compulsive | 1.414±0.500 | 1.349±0.501 | -0.065 | -4.13 | 0.0000 | -0.146 |
| Interpersonal Sensitivity | 1.291±0.438 | 1.231±0.434 | -0.061 | -4.08 | 0.0000 | -0.144 |
| Depression | 1.260±0.414 | 1.227±0.439 | -0.033 | -2.41 | 0.0162 | -0.085 |
| Anxiety | 1.231±0.395 | 1.198±0.404 | -0.033 | -2.51 | 0.0123 | -0.088 |
| Hostility | 1.237±0.421 | 1.196±0.409 | -0.041 | -2.81 | 0.0051 | -0.099 |
| Phobic Anxiety | 1.129±0.309 | 1.110±0.313 | -0.018 | -1.62 | 0.1051 | -0.057 |
| Paranoid Ideation | 1.217±0.392 | 1.174±0.382 | -0.043 | -3.10 | 0.0020 | -0.109 |
| Psychoticism | 1.172±0.346 | 1.139±0.340 | -0.034 | -2.86 | 0.0044 | -0.101 |
| Additional Items | 1.304±0.447 | 1.272±0.448 | -0.033 | -2.21 | 0.0271 | -0.078 |
| total | 112.887±32.757 | 109.440±34.011 | -3.447 | -3.21 | 0.0014 | -0.113 |
| GSI | 1.254±0.364 | 1.216±0.378 | -0.038 | -3.21 | 0.0014 | -0.113 |

**Supplementary Notes**

**S1. De-identification procedure**

Personal names were removed from the raw export and replaced by an 8-character identifier derived as the first eight hexadecimal characters of the MD5 hash of the original name. This allows the same firefighter to be matched across years within the analytical dataset without preserving any identifying information. City names were recoded to City A and City B; the assignment is arbitrary and the original city identifiers were not retained in the analytical file. Date of birth, address, employee identifier and any other potentially identifying field present in the original export were dropped before analysis.

**S2. Software environment**

All analyses were carried out in Python 3.11 with pandas 2.x, NumPy 1.26, SciPy 1.13, statsmodels 0.14 and matplotlib 3.8. The full analysis script is provided in the file analysis_code.py accompanying this submission. Re-running this script against the de-identified CSV reproduces every numerical value reported in the main text and supplementary tables, and regenerates all figures.

**S3. Sensitivity analyses**

We re-ran the year-on-year trend analysis excluding (a) the 2021 sample and (b) the 2025 sample to check whether the trend is driven by either edge of the observation window. Excluding 2021 alone reduced the magnitude of the year coefficient on total score from −4.12 to −3.50 (still p < 0.001). Excluding 2025 alone reduced it to −3.86. Excluding both, the coefficient is −3.30 (p < 0.001). In all sensitivity variants the direction and statistical significance of the trend are unchanged, but the magnitude is meaningfully smaller, consistent with the interpretation given in the main text that part — but not all — of the apparent decline reflects features of the screening programme rather than genuine cohort-level improvement.

**S4. Items handled as missing**

Sixty-one records were excluded for implausible age (< 18 years; mostly age = 0). Two hundred and thirty-eight within-year duplicates were collapsed to a single record by retaining the first occurrence. One-hundred-and-seventy-one records had an education entry of "Other" which we treated as missing for the purpose of the multivariable regression; the GSI distribution within this subgroup did not differ meaningfully from the rest of the sample.
